# The Wants of the Hop-Picking Mission

**Published:** 1902-08-30

**Authors:** 


### Editor's Letter-Box.

[Our Correspondents are reminded that prolixity is a great bar to publication, and that brevity of style and conciseness of statement greatly facilitate early insertion.]

### THE WANTS OF THE HOP-PICKING MISSION.

The Rev. J. E. Bevington-Jones, Hon. Secretary Hop-Picking Mission Committee, Rector of Mereworth, and Rural Dean, writes:—

"In about a fortnight's time the annual invasion of Mid and West Kent will take place. The hop-pickers are nearly all Londoners, and of the poorest. We try to help them in their almost necessarily rough-and-ready surroundings, where they are without many of the necessities of an even fairly decent life, let alone any of its comforts.

"'We are sending them,' he says, '25 experienced lady workers, and 13 trained nurses, to work from 14 centres, opening small hospitals for children, dispensaries for first aid, &c., coffee-stalls and barrows, and distributing illustrated papers, magazines, &c.,' as well as opening reading and writing rooms in the evenings and on Sundays.

"May I again appeal to your readers, as by your kindness I have done in past years, for money, nursing requisites, and literature of all kinds.

"All our officers and workers are volunteers; no one is paid, only out-of-pocket expenses are refunded. The working expenses of the committee average about 6 per cent. £5 pays for a nurse or worker.

"Subscriptions may be paid into Messrs. Child's Bank, 1 Fleet Street, E.C., or may be sent to me at Mereworth Rectory, Maidstone, where all literature should be sent."

